# Neurobiology and sleep disorders in cluster headache

**DOI:** 10.1186/s10194-015-0562-0

**Published:** 2015-08-20

**Authors:** Mads Christian Johannes Barloese

**Affiliations:** Danish Headache Center, Glostrup Hospital, Nordre Ringvej 57, Glostrup, DK-2600 Denmark; Department of Clinical Physiology, Frederiksberg and Bispebjerg Hospitals, Nordre Fasanvej 57, DK-2000 Frederiksberg, Denmark

## Abstract

Cluster headache is characterized by unilateral attacks of severe pain accompanied by cranial autonomic features. Apart from these there are also sleep-related complaints and strong chronobiological features. The interaction between sleep and headache is complex at any level and evidence suggests that it may be of critical importance in our understanding of primary headache disorders. In cluster headache several interactions between sleep and the severe pain attacks have already been proposed. Supported by endocrinological and radiological findings as well as the chronobiological features, predominant theories revolve around central pathology of the hypothalamus. We aimed to investigate the clinical presentation of chronobiological features, the presence of concurrent sleep disorders and the relationship with particular sleep phases or phenomena, the possible role of hypocretin as well as the possible involvement of cardiac autonomic control. We conducted a questionnaire survey on 275 cluster headache patients and 145 controls as well an in-patient sleep study including 40 CH-patients and 25 healthy controls. The findings include: A distinct circannual connection between cluster occurrence and the amount of daylight, substantially poorer sleep quality in patients compared to controls which was present not only inside the clusters but also outside, affected REM-sleep in patients without a particular temporal connection to nocturnal attacks, equal prevalence of sleep apnea in both patient and control groups, reduced levels of hypocretin-1 in the cerebrospinal fluid of patients and finally a blunted response to the change from supine to tilted position in the head-up tilt table test indicating a weakened sympathoexcitatory or stronger parasympathetic drive. Overall, these findings support a theory of involvement of dysregulation in hypothalamic and brainstem nuclei in cluster headache pathology. Further, it is made plausible that the headache attacks are but one aspect of a more complex syndrome of central dysregulation manifesting as sleep-related complaints, sub-clinical autonomic dysregulation and of course the severe attacks of unilateral headache. Future endeavors should focus on pathological changes which persist in the attack-free periods but also heed the possibility of long-lived, cluster-induced pathology.

## Introduction

One of the earliest descriptions of cluster headache (CH) in medical literature is provided by 17th century Dutch surgeon and mayor of Amsterdam Nicolaes Tulp (Famously depicted in Rembrandt’s The Anatomy Lesson of Dr Nicolaes Tulp.) [1]:

“. . . in the beginning of the summer season, [Isaak van Halmaal] was afflicted with a very severe headache, occurring and disappearing daily on fixed hours, with such an intensity that he often assured me that he could not bear the pain anymore or he would succumb shortly. For rarely it lasted longer than two hours. And the rest of the day there was no fever, nor indisposition of the urine, nor any infirmity of the pulse. But this recurring pain lasted until the fourteenth day . . .”

There can be little doubt that the patient described was suffering from arguably the most painful condition known in medicine [2]. With its stereotypical presentation and the ease with which the diagnosis can be made (even based on a short historical account), it may seem puzzling how CH can remain underdiagnosed, undertreated and so enigmatic in nature. Accounts of the predictable attack patterns and how oxygen, almost by miracle, alleviates the headache have fascinated many, but true progress in our understanding of the disorder is lacking.

CH is a primary headache disorder belonging to the diagnostic group known as the trigeminal autonomic cephalalgias (TAC’s) [2]. These headaches share the common features of short-lasting, severe, strictly unilateral pain in the distribution of the first division of the trigeminal cranial nerve. Attacks last from 15 to 180 min and simultaneous activation of the trigeminal autonomic reflex produces the characteristic cranial, autonomic, accompanying symptoms (Table 1) [2]. Systemic manifestations are also present as patients almost universally become agitated, restless and, as opposed to migraineurs, do not experience worsening of the pain during movement, perhaps rather relief. Uniquely, CH patients describe their attacks to exhibit remarkable circadian and annual periodicity . The attacks are described to strike at predictable times of the day and the clusters (bouts) of these at specific times of the year. However, there is no consensus as no two studies have ever reached identical conclusions concerning these patterns [3–7].

**Table 1:** Diagnostic criteria for cluster headache according to the ICHD-2 [2]

Cluster headache
A. At least 5 attacks fulfilling criteria B-D
B. Severe or very severe unilateral orbital, supraorbital and/or temporal pain lasting 15–180 min if untreated
C. Headache is accompanied by at least one of the following:
1. Ipsilateral conjunctival injection and/or lacrimation
2. Ipsilateral nasal congestion and/or rhinorrhoea
3. Ipsilateral eyelid oedema
4. Ipsilateral forehead and facial sweating
5. Ipsilateral miosis and/or ptosis
6. A sense of restlessness or agitation
D. Attacks have a frequency from one every other day to 8 per day
E. Not attributed to another disorder
Episodic cluster headache
A. Diagnostic criteria A-E for cluster headache
B. At least two cluster periods lasting 7–365 days and separated by pain-free remission periods of ≥ 1 month
Chronic cluster headache
A. Diagnostic criteria A-E for cluster headache
B. Attacks recur over > 1 year without remission periods or with remission periods lasting < 1 month.

Diagnostically, CH exists in two forms: an episodic (ECH) and a chronic variant (CCH), distinguished by the duration of the attack-free periods [8]. This division is diagnostic and there is no evidence for a clear reflection of prognosis, response to treatment or causative pathology. The social impact of CH is considerable [9] and it is associated with sizeable direct and indirect economic consequences [10]. Consequently, endeavors to uncover the pathological mechanisms behind this disabling headache have intensified over the past two decades; regrettably, the challenges associated with investigating the transient, severe pain attacks accompanied by agitation have slowed progress. Firstly, it is difficult to capture the attacks and clusters, secondly it may be difficult for the patients to remain still, complicating measurements. Lastly, provoking the attacks may cause changes obscuring the findings themselves.

### The chronobiological nature of cluster headache

Chronobiology is the study of biological rhythms. In humans, by far the most noticeable rhythm is the diurnal sleep-wake rhythm which roughly follows a 24-h pattern [11]. This, along with a multitude of other changes in the organism, is evoked by the light and dark periods of day and night – a consequence of the 24 h. rotation of the earth around its own axis. These periods and other *Zeitgebers* entrain the master circadian clock located in the hypothalamic suprachiasmatic nucleus (SCN) [11]. Through the release of melatonin from the pineal gland the SCN commands the overall rhythm of the organism.

CH is arguably the headache disorder which demonstrates the strongest chronobiological characteristics. The attacks themselves have been described to be mostly related to (nocturnal) sleep and to follow specific rhythms that often provide a high degree of predictability [7, 12]. Nonetheless, numerous unresolved issues in our understanding of the pathophysiology and the relation to circadian and sleep-wake regulation remain. The pioneers of CH chronobiology described the circadian and annual rhythmicity [5, 6], but it is unclear whether the biphasic signal of cluster occurrence in ECH is a function of the solstices, the equinoxes or something else [3, 7]. Further, it appears that the circadian periodicity may be influenced by cultural factors but the precise mechanisms remain obscured [3, 6, 7].

### Cluster headache is a sleep-related headache

For reasons which are partly unknown headache and sleep share an especially close relationship, as evidenced by a dense anatomical and physiological overlap in the central nervous system (CNS) [13] but also by a high degree of co-occurrence of sleep problems and headache [14]. To reflect this interesting, yet poorly understood interaction, migraine, hypnic headache, chronic paroxysmal hemicranias and CH are all classified as “sleep-related headaches” in the International Classification of Sleep Disorders [15].

Inspired by anecdotal and clinical experience, sleep studies in CH have been conducted but results are diverging [12]. Anecdotally, patients awakened by nocturnal attacks often lucidly recall dreams and typically report that these occur one to two hours after falling asleep. These sporadic observations have led to the belief that nocturnal attacks of CH are temporally related to the rapid-eye-movement (REM) sleep phase, the first of which typically occurs roughly one hour after sleep onset. The possible connection with REM-sleep [16–18] and sleep apnea [17, 19–22] is based on relatively small studies which are mostly uncontrolled. It appears that while a temporal association between individual CH attacks and nocturnal sleep (but not necessarily REM-sleep) is evident, little is known about the specifics of this link.

### Hypocretin - a neuropeptide with a potential role in CH pathology

A theory of hypothalamic involvement predominates in CH pathology. The strongest evidence in favor of this are radiological findings demonstrating specific and exclusive hypothalamic activation during CH attacks [23] as well as increased hypothalamic grey matter volume in CH patients [24]. Hypothalamic involvement is further evidenced by the efficacy of deep brain stimulation (DBS) of the posterior hypothalamus in medically refractory CH [25, 26], endocrinological changes (reduced melatonin, testosterone, noradrenaline among others) [27] and the chronobiological features of the disorder (rhythmicity of the attacks and clusters) [28].

**Fig. 1:**
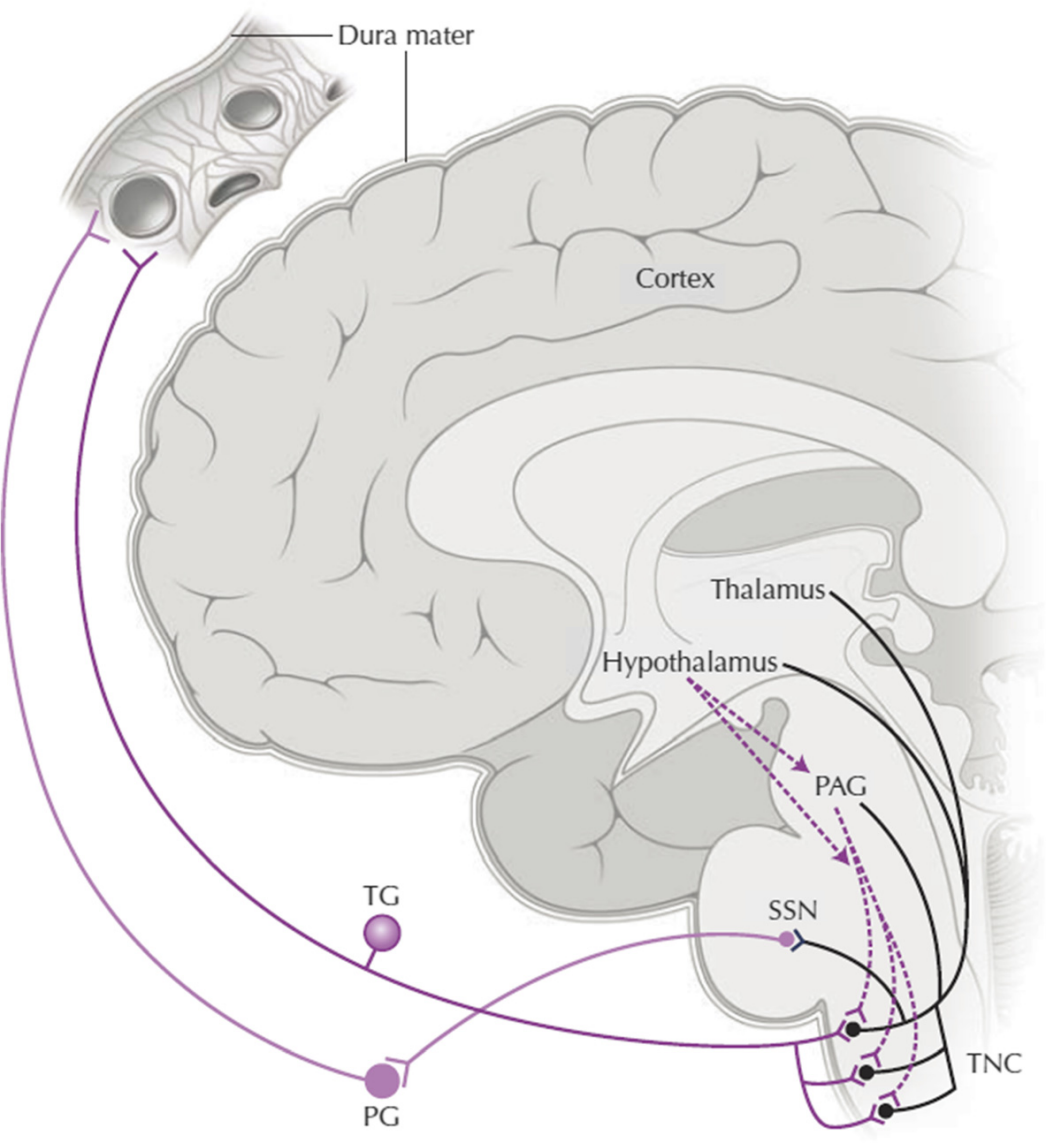
Schematic depiction of the trigeminal-autonomic reflex and related areas. Sensory afferents from cranial structures synapse in the trigeminal nucleus caudatus. Input is relayed to the brainstem and higher structures including the periaqueductal grey (PAG) and hypothalamus. Likewise, descending modulatory hypocretinergic connections are received from the hypothalamus. SSN – superior salivatory nucleus, TNC – trigeminal nucleus caudalis (trigeminal complex), PG – ptyrogopalatine (sphenopalatine) ganglion, TG – trigeminal ganglion. With permission from Holland et al. 2009 (Springer)

Hypocretins (HCRT) 1 and 2 (also known as orexin A and B) are neuropeptides produced by 10–20,000 neurons in the lateral and perifornical areas of the hypothalamus [29]. Caused by the complete loss of HCRT neurons, perhaps by an autoimmune process, the HCRT concentration in cerebrospinal fluid (CSF) of patients suffering from narcolepsy with cataplexy is low to undetectable [30]. The HCRT-1 and −2 receptors are G-protein coupled, and located widely throughout the neuroaxis [31]. HCRT-1 binds to both the HCRT-1 and −2 receptor with equal affinity whereas HCRT-2 binds to the HCRT-2 receptor with ten times greater affinity [31]. Crucial for the normal function of arousal control, sleep regulation, homeostatic maintenance and possibly pain processing [29], the HCRTs may be involved in CH pathology by way of a polymorphism of the HCRT-2 receptor gene [32–35] or an otherwise related mechanism, possibly involving descending connections from the hypothalamus to brainstem circuits involved in trigeminal nociception (Fig. 1).

### Systemic manifestations of hypothalamic dysfunction

The aforementioned findings of central involvement would suggest that systemic manifestations of central autonomic dysregulation may be present. In CH, ictal involvement of the cranial autonomic nervous system is undeniable and an inherent feature of the disorder. It is observable to the surroundings in the form of the accompanying symptoms that form part of the diagnostic criteria. These symptoms result from activation of the trigeminal autonomic reflex producing hyperfunction of the parasympathetic division and hypofunction of the sympathetic [36]. Peripheral stimulation of the pterygopalatine ganglion (PG) may induce cluster-like attacks [37]. Systemically, reports of changes in the electrocardiogram (ECG) and ictal bradycardia [38–42] suggest that general autonomic control *is* affected in CH. However, results are contradictory [38, 39, 41–50]. A relatively unobtrusive method for studying the function of central regulation of the cardiovascular system is by analysis of heart rate variability (HRV), allowing dissection of the contributions of the parasympathetic and sympathetic divisions of the ANS. Being a relatively new method of characterizing autonomic function, studies of spectral analysis in CH are rare [43, 45].

## Hypothesis and aims

A detailed review of the mechanisms and interactions described above has been published [12] (study I) and it serves as the pillar-stone for the studies encompassed in the present thesis. Generally, the thesis is based on a theory of central pathology as the cause of CH. We hypothesize that this central pathology revolves around dysfunction of hypothalamic nuclei, producing conditions in which the painful attacks can arise or perhaps that these nuclei function as a “cluster generator”. This gives rise to the characteristic triad of extreme pain in the first division of the trigeminal nerve with accompanying autonomic symptoms, impaired sleep regulation and chronobiological rhythmicity. This dysregulation manifests itself as a complex, bidirectional relationship with sleep involving neuronal circuits in the overlap between headache pathology and the physiological regulation of sleep, as reviewed in [12]. Thus, the hypocretinergic system is affected in CH either as an intrical part of disease mechanisms or as an epiphenomenon, reflecting overall hypofunction of hypothalamic nuclei and descending, antinociceptive projections. It is also likely that central autonomic control in CH patients is affected. This may be an essential factor in the triggering of attacks or may reflect generally altered function of central autonomic command.

The overall aim of the project was to investigate the interaction between CH and sleep and the neuro- and chronobiological features of the disorder. Specifically:

A. To investigate sleep quality, chronotype, triggers and chronobiological features of CH in a large population of well characterized patients [28].
B. To study the macrostructure of sleep as well as breathing parameters, arousals, periodic limb movements (PLM’s), limb movements (LM’s) and spontaneous CH attacks’ relationship to sleep in general and specific sleep phenomena [51].
C. To measure the HCRT-1 concentration in CSF from CH patients during an active bout to investigate whether levels of HCRT reflects activity of CH [52].
D. To characterize overall autonomic function, as measured in a head-up tilt-table test (HUTT), in a population of CH patients during the active bout of their headache disorder and compare this to matched, healthy controls [53].

## Review

### Materials and methods

**Fig. 2:**
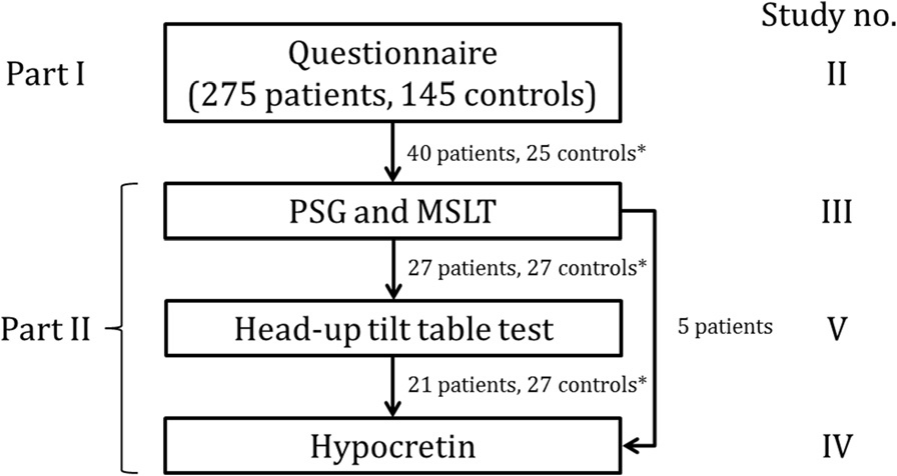
Study design and patients included in the final analysis at each stage. Hypocretin was measured in cerebrospinal fluid obtained via spinal tap. *Control groups were different in all of the investigations. PSG – polysomnography, MSLT – multi-sleep latency test

The data forming the basis of analyses in this thesis was collected at the Danish Headache Center between winter 2012 and spring 2014. A total of 275 patients and 223 controls in four groups participated in four different studies (Fig. 2). These were approved under the same protocol by the ethics committee of the Capital Region of Denmark (H-2-2012-016) and all patients and controls gave their written consent in accordance with the Declaration of Helsinki.

The entire project was composed of two parts (Fig. 2): A questionnaire and interview-based study aiming to include as high a number of CH patients as possible (part 1) and an in-hospital clinical investigation including 40 CH patients (part 2). All patients completed the same questionnaire and interview which also provided the clinical characterization of the 40 patients participating in part 2.

### Part one: Cross-sectional questionnaire and interview

#### Patients

Patients diagnosed with ECH or CCH were recruited from a register of present and former contacts at the Danish Headache Center. Additionally, a notice was posted in the newsletter and on the website of the Danish patient organization for CH. Other headache clinics in Denmark were contacted and patients receiving treatment at these clinics were referred to the study if they were interested. Patients were included if they were between 18 and 65 years old, had been diagnosed with ECH or CCH (ICHD-II criteria) and were able to tell CH-attacks apart from other types of headache. Patients were excluded if they had been diagnosed with another primary or secondary chronic headache (≥14 days/month) or did not speak and understand Danish. All patients’ diagnoses were verified by headache specialists at the Danish Headache Center.

#### Controls

Controls were recruited via notices posted on the internet and in work places and sport clubs in the Capital Region of Denmark. Controls were matched for sex and age and were required to be between 18 and 65 years old and headache-free (≤1 day of headache/month). They were excluded if they had severe symptoms of sleep disorders.

#### Questionnaire

A questionnaire composed of already validated sections (Pittsburgh Sleep Quality Index (PSQI), Morningness-Eveningness Questionnaire (MEQ)) and new questions concerning headache characteristics, life style, impairment and others developed by MB and RJ was sent to patients and controls. In the final phase of development the questionnaire was assessed for content and face validity by an expert panel comprised of 10 senior headache experts (unpublished data). They were asked to evaluate the questions: Are the instructions clear, is the questionnaire coherent, are the questions relevant, is the questionnaire logically divided into parts, are the questions formulated clearly. The questionnaire was also tested with a randomly selected population of 10 CH patients representing different ages, sexes and diagnoses (ECH and CCH) and comments and suggestions were implemented. The paper questionnaire contained 362 questions in 7 sections: Headache diagnosis, headache burden, treatment, sleep, work, lifestyle habits, and physical activity. It was estimated that it took the patients roughly one hour to complete the questionnaire. Following completion of the questionnaire, answers were verified and ambiguities ruled out by a structured interview conducted by a physician or a trained medical student. If the patient or control invited to participate did not respond within 21 days they were contacted again by letter and/or telephone and encouraged to complete the questionnaire.

The PSQI [54] is a validated [55] 19-item measure of subjective sleep quality during the past month. The sum of seven component scores provides a global score, a higher value reflecting poorer sleep quality. A global score greater than 5 yields a high sensitivity and specificity in distinguishing “good and poor sleepers” [54].

The MEQ [56] uses 19 multiple-choice items to chronotype patients into five categories: Definite morning, moderate morning, intermediate, moderate evening, definite evening. The questionnaire has been validated in middle aged populations [57]. Chronotype reflects at what time of the day a person is active or inactive, often reduced to sleeping habits only, i.e. “lark” or “owl”.

To characterize rhythmicity of attacks and clusters, patients were asked to report the hours of the day or the months of the year where these were most likely to occur. They also had the option to report no rhythmicity. For further details please see [28].

### Part two: In-hospital investigation of sleep, hypocretin and autonomic function

Forty patients underwent clinical examination, routine blood sampling, polysomnography (PSG) and multi-sleep latency test (MSLT). 29 of the patients completed the tilt-table test and 27 the spinal tap procedure. All investigations were done at Glostrup Hospital, Departments of Neurology, Neurophysiology and Diagnostics, and at the Coordinating Research Centre, Dept. of Clinical Physiology and Nuclear Medicine, Frederiksberg Hospital.

#### Patients

Patients for part 2 were recruited as described above and were investigated during the active cycle of the headache disorder (1–8 attacks/day over the past week) and at least two weeks into the bout. Exclusion criteria were other chronic primary or secondary headaches and serious somatic or psychiatric illness. If patients were on prophylactic medication this was kept stable for at least seven days prior to investigation. Patients would attempt to treat their attacks using oxygen but were allowed to use other medication such as injectable or nasal triptans, as a rescue.

#### Controls

Three separate populations of controls were used:

##### Sleep investigation

For study III 25 controls with an age, sex and BMI-makeup similar to the patients were included. Controls were recruited through www.forsoegsperson.dk, a website for healthy volunteers, and did not suffer from chronic headaches, sleep disorders or any other health problem (one control had mild, controlled hypertension and hypercholesterolemia) as concluded by interview, examination and questionnaires. For further details please see [51].

##### Hypocretin samples

For study IV 27 healthy controls without headache-, sleep- or other neurological disorders were included from a prior study [58]. Twelve of the subjects were recruited through advertisement for healthy volunteers (www.forsoegsperson.dk) and clinical and neurological examination by a physician was conducted. The remaining 15 subjects were referred for subjective sleep-related complaints but were found healthy by experienced sleep specialists by interviews, normal clinical and neurological findings, PSG and MSLT. Controls were not matched according to age or sex as these factors may not influence HCRT-levels [59].

##### Head-up tilt table test

For study V 27 controls matched according to age, sex, BMI were included. All controls were interviewed to ensure they were healthy and did not suffer from disorders including primary headaches. For further details please see [53].

### Methods

#### Polysomnographic recordings

Recordings took place during admission at the Danish Center for Sleep Medicine and Department of Neurology at Glostrup Hospital, Denmark. PSG recordings were performed and scored in accordance with the AASM standard [15]. Final assessment and possible sleep disorder diagnoses were made by senior doctors, specializing in sleep medicine according to the ICSD-2 [15]. Two nights of PSG-recordings were made for patients and one night for controls. MSLT was done after the last night of sleep recording. Additionally, for detailed methods please see [51].

#### Measurement of cerebrospinal fluid hypocretin-1

10 mL CSF was collected by the spinal tap procedure in the attack-free state between 08.00 and 12:00. Hypocretin-1 was analyzed in crude CSF by radioimmunoassay from Phoenix Pharmaceuticals (Belmont, CA, USA). We used the same methodology as in a previous study [58]. Assay quality was monitored by the internal positive control sample included in the assay kit. Previously used groupings of CSF intervals for HCRT-1 concentrations (low (≤110 pg/ml), intermediate (>110 ≤ 200 pg/ml), and normal (>200 pg/ml)) were not strictly applied in this sample as these are most relevant in the diagnosis of narcolepsy [30, 60, 61]. For detailed methods please see [52].

### Head-up tilt table test

All tests were performed in the fasting state between 08.00 and 14.00 h. at standard room temperature. After 10 min. of supine rest, baseline data were acquired during a further 10 min. of rest, where the subjects refrained from speaking and from unnecessary movements. The subjects were then loosely strapped to an electrically driven tilt table and tilted to a 60° head-up position (HUT) within 10 s. and stayed in this position for at least 10 min. if intolerable symptoms did not appear. RR-intervals and blood pressure (BP) were measured continuously from a bipolar 2-channel ECG and by Finometer equipment (Finapres Medical Systems BV, Amsterdam, The Netherlands), respectively. Baseline values of HR, SBP and diastolic blood pressure (DBP) were calculated as mean values from the 30 s. preceding tilt in the supine position and during 30 s. obtained in the 5th and 10th min. of HUT. Analysis of HRV was performed according to current guidelines [62] using share-ware (Kubios, vers. 2.0, kubios.uef.fi). For detailed methods please see [53].

### Data and statistics

Characterization of the patients for all four studies was based on the questionnaire and interview. A CH index was calculated by the following equation: A*ttacks per day x hours per attack x days per cluster x clusters per year* and is a measure of the total time/year that the patient has cluster headache. In this specific calculation, for CCH patients, number of clusters/year was set at one and cluster duration was set at 365 days. The motivation for calculating this index is to provide a single number which reflects the amount of headache, as this is sometimes not completely clear, taking into consideration clusters, cluster duration, attacks and attack duration. Further, we wanted to characterize the patients beyond the dichotomy of episodic-chronic as some episodic patients in fact may experience far more headache than some chronic.

Apart from diagnosis (ECH, CCH) and sex, the patients were stratified based on self-reported characteristics including: “Annual rhythmicity” – clusters occur at the same time each year, “diurnal rhythmicity” – attacks occur at the same time each day, “sleep attacks mostly” – the patient reports that the majority of attacks occur during sleep, “sleep *and* awake or mostly awake” – the patient reports no predominance of attacks during sleep, attacks during PSG monitoring and whether the patients primarily suffered attacks during sleep or both during sleep and wake. The data gathered in the questionnaire and interview study allowed for even finer distinction of patients and an analysis of the exact times patients reported attacks was made: 08.00-21.00, 22.00-07.00. BMI was calculated from self-reported height and weight (BMI = kg/m^2^). Tobacco “pack-years” was calculated as no. of cigarettes (or equivalent) smoked daily/20 x no. of years smoking.

SAS 9.3 or 9.4 was used for all statistical analyses. *P* < 0.05 was considered statistically significant. Levene’s test for homogeneity was used to check variance. *T*-test was used to compare two groups and ANOVA for comparison between more than two groups. Bonferroni corrections were applied in the analysis of PSQI and in the comparison of diagnostic subgroups in HCRT-analysis. Chi-square test was used to calculate differences in sex composition, smoking status, MEQ-groups, laterality and prevalence of sleep apnea. Linear regression was used to describe the association between daylight hours and cluster occurrence and the CH index and PSQI. The Wilcoxon rank sums test was used for non-parametric data.

In the analysis of PSG data, the first PSG in patients and controls were compared using unpaired statistics. All comparisons between patients and controls only used the first night of data for patients to ensure that data was comparable. For consistency, the patients’ first and second night of recording were compared with paired statistics.

In the analysis of HUT data, HR and BP and changes in the time domain were analyzed as changes in percent to account for baseline values.

## Results

### Questionnaire: sleep and chronobiology

#### Response rate and clinical characteristics

The response rate for both patients (57.2 %) and controls (54.5 %) was within accepted and expected ranges [14]. 275 patients and 145 age and sex-matched controls completed the questionnaire and interview (Table 2). For further details please see [28].

**Table 2:** Demographics for patients in the questionnaire study.

	N	Diagnosis	Age yrs	Sex, %M	BMI
Patients	275	187E/88C	47.9 (11.1)	69.5	25.5* (4.2)
Controls	145		46.9 (12.9)	69.7	24.5* (3.5)

Data are expressed as means (SD). *P < 0.05. E – episodic, C – chronic

While the primary focus of the study was to investigate chronobiological features, some findings were of a more clinical nature. As expected, on average, chronic patients scored 7-fold higher on the CH-index (935.6 ± 890.2) compared to episodic (135.6 ± 204.4). This is a considerable difference and it should be noted that there is a huge span and some ECH patients in fact scored very highly (Range: ECH = 0.35-1428, CCH = 60-5096). Interestingly, a difference between these two groups in attack duration *with* treatment was found, CCH patients suffering longer attacks (44 vs. 32 min., *P* = 0.0052).

#### Chronobiology

As expected far more ECH patients than CCH reported annual rhythmicity (65.2 % vs. 36.4 %, *P* < 0.0001) [28]. However, while there was a difference in the prevalence of annual rhythmicity, there was no difference in the prevalence of circadian rhythmicity between the two patient groups (ECH: 83.4 % vs. CCH: 79.5 %, *P* = 0.4332). Interestingly, we also found that patients exhibiting one type of rhythmicity were more likely to exhibit the other.

Eighty percent of patients reported that nocturnal sleep (as opposed to napping) was an attack trigger. Only 1/3 of patients reported that napping triggered attacks. Patients with diurnal rhythmicity more frequently reported sleep as a trigger compared to patients without diurnal rhythmicity (*P* < 0.0001).

Characteristic patterns were discovered in the analysis of attack incidence during the 24 h. of the day. By far the most reported time for nocturnal attacks was 02.00 h. During the day, the most frequently reported time for attacks was 16.00 h. Three low points, roughly coinciding with meal times, are noted – 09.00, 12.00 and 18.00. From 18.00 and onwards attack incidence climbs steadily towards its peak at 02.00.

Looking at annual cluster incidence (or worsening of attacks for CCH) a clear pattern of betterment during the late spring – summer – early autumn was identified. A regression analysis comparing cluster incidence and daylight hours was highly significant (*P* = 0.0002).

#### Sleep quality

Patients scored significantly higher on the PSQI (8.4 vs. 4.0, *P* < 0.0001) compared to controls indicating a poorer subjective sleep quality [28]. A negative relationship between time passed since last attacks and PSQI score was identified (*P* < 0.0001). However, even one year after patients suffered their last attack, PSQI remained above the pathological cut-off at 5. In a subgroup analysis the same trend was identified in the following groupings:

- Patients reporting sleep as a trigger (*n* = 220).
- Patients reporting no sleep-trigger (*n* = 55).
- Patients reporting exclusively attacks between 22.00-07.00 (*N* = 72).
- Patients reporting attacks during all 24 h of the day (but not exclusively 22.00-07.00) (*N* = 203).
- Patients reporting exclusively daytime attacks (08.00-21.00) (*N* = 18). Overall these patients still reported a high PSQI score (7.1), despite not suffering nocturnal attacks.

### In-hospital clinical investigation

#### Sleep in CH

Forty patients and 25 age- and sex-matched controls contributed a total of 99 nights for analysis (Table 3). Findings included lower REM-density (17.3 vs. 23.0 %, *P* = 0.0037) and latency (2.0 vs. 1.2 h., *P* = 0.0012) in patients compared to controls [51]. Apart from the affected REM-sleep, macrostructurally there was no difference between patients and controls except for lower efficiency (77 vs. 87 %, *P* = 0.0026) and longer sleep latency (0.56 vs. 0.18 h, *P* = 0.0057). There was no temporal association between REM-sleep, any other sleep stage or particular sleep-related events, including apneas and desaturations and observed, spontaneous nocturnal attacks (*N* = 45). Furthermore, patients had far fewer arousals (7.3 vs. 14.1, *P* = 0.0030). As opposed to all previous studies no difference in prevalence of sleep apnea in patients vs. matched controls (38 vs. 34 %, *P* = 0.64) was identified. However, average AHI in patients was numerically higher (AHI 10.75 vs. 4.93).

**Table 3:** Clinical characteristics. Attack duration is for treated attacks. Bout duration and bouts/year only includes ECH

		N	Diag.	Sex %M	Age (yrs.)	BMI	CH duration (yrs.)	Attack duration (min.)	Attacks/day	Bout duration (wks.)	Bouts/year
PSG & MSLT	Patients	40	21E 19C	73	44.2 (11.2)	25.7 (4.0)	11.2 (7.4)	35.0 (33.1)	4.6 (1.9)	12.3 (20.3)	1.7 (1.1)
	Controls	25		64	47.6 (12.1)	24.2 (3.4)					
HCRT	Patients	26	14E 12C	66	44.3 (11.5)	24.9 (3.8)	10.3 (5.9)	27.2 (18.2)	4.3 (1.8)	14.2 (8.8)	1.6 (0.9)
	Controls	27		44	32.4 (9.5)						
HUT	Patients	27	14E 13C	70	44.9 (11.3)	25.4 (3.8)	11.3 (7.8)	37.2 (37.8)	4.4 (1.8)	12.7 (9.2)	1.7 (1.2)
	Controls	27		70	49.0 (8.3)	24.4 (2.7)					

Data is presented as means (SD). PSG – polysomnography, MSLT – multi-sleep latency test, HCRT – hypocretin, HUT – head-up tilt table test, E – episodic, C – chronic

**Fig. 3:**
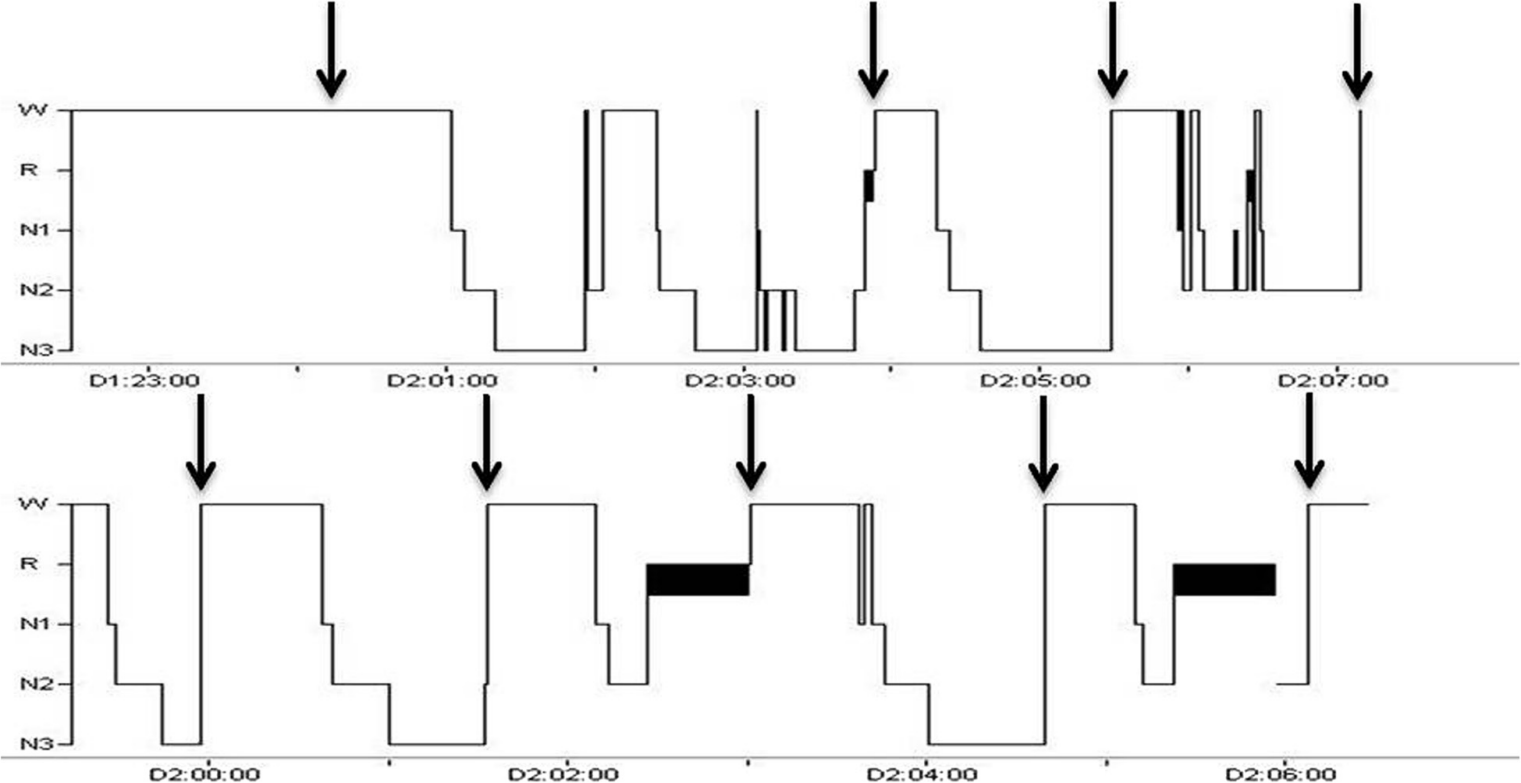
Hypnograms from night 1 (top) and 2 (bottom) from a patient suffering nine spontaneous CH attacks (arrows) during recordings. As is seen, the attacks occur in stages W, REM, N2 and N3 at remarkably regular intervals. With permission from Barloese et al. 2014 (Wiley) [51]

In one patient a striking rhythmicity of nocturnal attacks was noticed (Fig. 3). This patient suffered a total of nine attacks during the two nights of recording. There was no relation with any particular sleep stage, rather the attacks occurred roughly every 90 min. During night 1, at 02.00, the patient wakes up but without an attack.

#### Hypocretin

**Fig. 4:**
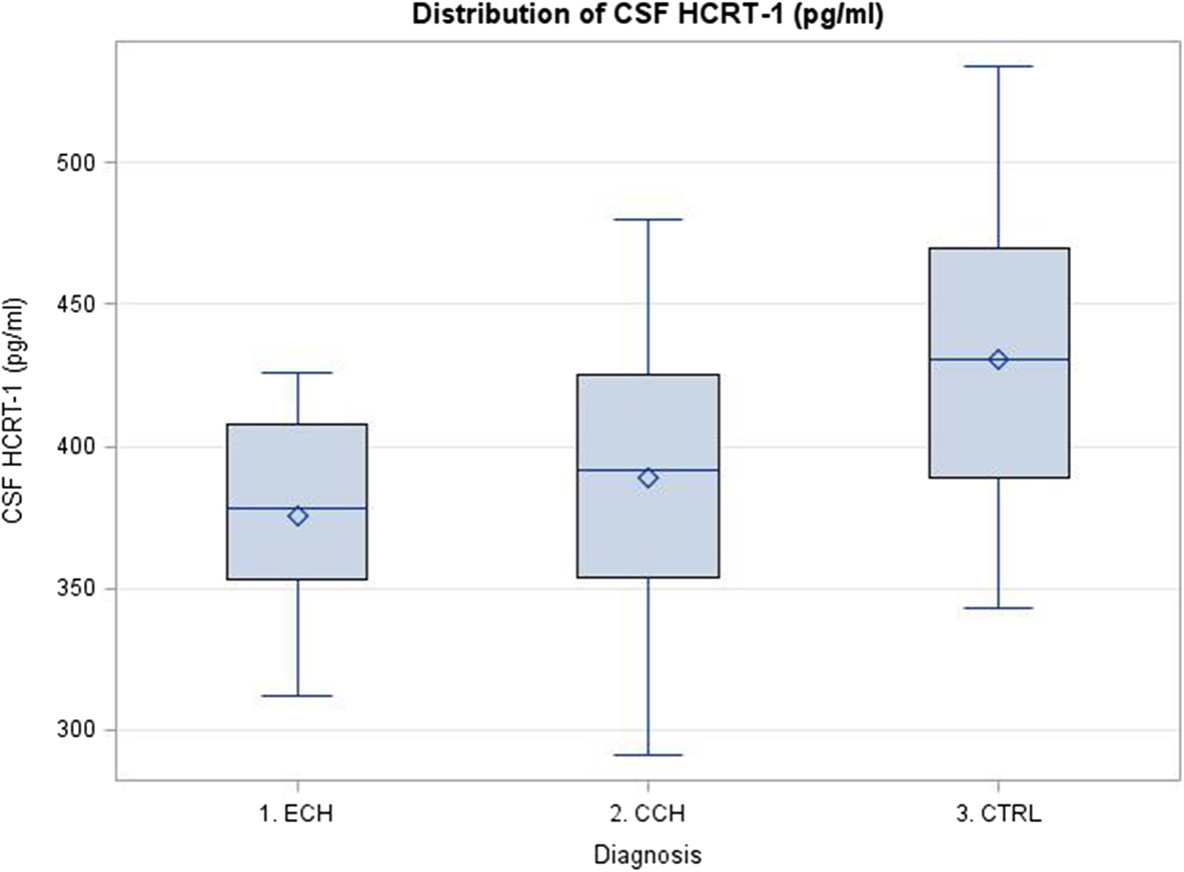
Hypocretin-1 levels in patients and controls. ECH – episodic cluster headache, CCH – chronic cluster headache, CTRL – control, HCRT-1 – hypocretin 1

In total 26 patient and 27 control samples of CSF were included in the HCRT-1 analysis (Table 3). A highly significant reduction of HCRT-1 levels in patients compared to controls was identified (382 vs. 431 pg/ml, *P* = 0.0004) [52]. Both subgroups of CH patients (ECH (375 pg/ml, *P* = 0.0005) and CCH (389 pg/ml, *P* = 0.0221)) were significantly reduced compared to controls (Fig. 4). There was no difference between the two subgroups of patients. However, on average, CCH patients had higher concentrations and the range and standard deviation was greater (CCH: 291-480 ± 60 vs. ECH: 312-426 ± 38). There were no significant differences in HCRT-1 concentrations between patients dichotomized according to rhythmicity and sleep attacks.

#### Autonomic function

There were no differences between patients and controls in standard cardiovascular responses to tilt including HR and BP. However, patients did present a higher BP throughout the test (Patients: 121–128/79–88 mmHg, controls: 107–115/56–68 mmHg, *p* < 0.01–0.0001) [63]. In both the non-linear and frequency domain analyses, patients presented a blunted response to tilt. These differences were significant in the analysis of normalized units (HF n.u. and LF n.u.) and the LF/HF ratio as well as the SD1/SD2 ratio. Within the CH population there were no differences when dichotomizing according to subdiagnosis, rhythmicity and sleep- or sleep and wake attacks [63].

## Discussion

In the investigations made over the past 30 years several theories about sleep and nocturnal CH attacks have been proposed including a strictly temporal connection with the REM phase of sleep [17, 64] and association between sleep apnea and CH [65]. While some case reports may seem to provide evidence of such a direct, causal relationships [66–68], it is likely that the interaction between sleep and CH is more complex, multifaceted and indirect in nature.

### Cluster headache is a chronobiological disorder

Cluster headache provides a unique opportunity to study a disorder which is present at very specific times of the year and then spontaneously remits and is, at least with regards to the headache, completely absent for long periods. Few other disorders show this kind of strong chronobiological features, and those that do have some interesting similarities with CH, such as the rare lithium responsive [69], recurrent hypersomnia Kleine-Levin syndrome [70]. While it is obvious and easy to establish that the headache attacks completely remit outside of the cluster period in the episodic subforms, it is unknown whether underlying pathology remains present, perhaps manifesting in unknown ways.

The present results suggest that there may be a long-lived dysfunction present, which the majority of the time manifests as poor sleep quality and at specific times of the year perhaps when entrainment by natural light cues is weaker, results in periodic disinhibition of the trigeminal autonomic reflex. The anatomical substrate for this interaction may be the complex brainstem and hypothalamic circuits where an overlap between headache and sleep exists [13]. In susceptible individuals, at times of the year when Zeitgebers change or are weaker, resultant cluster penetration may involve melatonin metabolism. Melatonin concentrations have been shown to be lowered and to exhibit a blunted nocturnal peak in CH patients regardless of cluster presence [71–74], and when administered prophylactically it may have a slight, positive effect on the clusters [75]. The strong association between cluster occurrence and the amount of daylight demonstrated in this study further strengthens a hypothesis of an intricate relationship between melatonin metabolism and CH. A possible connection with testosterone has also been suggested which may be especially interesting considering REM-sleep’s effect on this hormone [76] and recent findings suggesting it may positively affect antinociceptive signaling in the trigeminal complex [77].

A PSQI score above 5 gives a high sensitivity and specificity in distinguishing “good and poor sleepers” [54]. In the results presented here, sleep quality improves as time passes since the last attack but remains abnormal even one year after the last attack, implying a permanent or long-lived dysfunction in sleep regulatory mechanisms. It also means that it is not only the nocturnal attacks themselves that in a direct manner disturb the sleep of CH patients, since sleep quality remains poor even in the attack-free state. This is further evidenced by the fact that the 18 patients reporting exclusively daytime attacks also reported poor sleep quality. Subjective sleep quality may thus be an indirect measure of the presence of an underlying pathological mechanism, and should be investigated further. A systematic, detailed recording of sleep history may be useful in the clinical evaluation of patients and sleep quality could be used to evaluate this sub-threshold presence of the cluster.

The classification of patients as episodic or chronic presents some problems unique to CH. For example, current diagnostic criteria [2, 8] do not specify whether the patient should be completely off preventive medication to be classified as episodic. Further, many patients classified as episodic may be significantly more affected by the headache than some patients fulfilling the criteria for chronic CH. For research purposes, it seems that the usefulness of dichotomizing according to subdiagnosis may not be universal. These arguments were the main reason for the development of the CH-index which reflects the total time the patient has suffered from headache over the past year. As can be seen, the range is huge and there is a significant overlap between ECH and CCH. Although the calculation is subject to recall bias, the index still provides one number reflecting the amount of headache the patient has, taking into account attack frequency, attack duration and cluster duration. When based on prospective recordings the index may precisely and in a comparable manner reflect total headache burden.

### A complex, bi-directional relationship with REM-sleep

The connection between CH and REM-sleep is the subject of a long-running debate. Early studies indicated a temporal relationship which was supported by observations that CH attacks typically occur 60–90 min. after falling asleep, coinciding with the first REM phase. Several reports, including the present results, now show that there is no relation with REM-sleep for any of the subdiagnoses [78, 79]. However, it is clear that REM-sleep is affected in CH patients but the cause remains unclear. Firstly, it must be taken into consideration that the homeostatic pressure for sleep and REM-sleep is most likely affected in this patient group as a result of nocturnal awakenings. Secondly, considering the described overlap of sleep and headache, it seems likely that changes in hypothalamic and brainstem nuclei may directly or indirectly affect REM sleep. The ventrolateral grey and lateral pontine tegmentum, receives hypocretinergic input, and may be an area of interest in this regard [13]. Further, in the present results there was no difference in the macrostructural composition of sleep between patients and controls apart from a lower REM density. Serotonergic and noradrenergic activity leads to suppression of REM sleep [80, 81] and the noradrenergic locus coeruleus and the serotonergic dorsal raphe nucleus are areas of direct anatomical overlap between sleep regulation and headache [13]. With this in mind, the challenge is to dissect the contributions from a change in homeostatic pressure and a change in function of the hypothalamic and brainstem nuclei involved in the regulation of sleep and pathology of headache. At present, our understanding of these circuits and interactions is not sufficient to make precise conclusions. A single case report hints at a fascinating change in sleep patterns taking place before the cluster [82]. This finding, and the fact that subjective sleep quality is worse even outside of clusters in ECH patients suggests that CH is a syndrome in which changes first manifest as dysregulation of sleep and secondly as a destabilization of trigeminal nociceptive processing.

### Sleep disordered breathing is a common finding in men

Up to 24 % of middle-aged males exhibit sleep disordered breathing (AHI ≥ 5) [83] and the studies suggesting an increased prevalence of sleep apnea in CH are uncontrolled [17, 20, 22] except two [19, 65]. Further, in earlier studies it is not always clear whether patients were in active bout during investigation and one study investigating the same patients inside and outside of bout does not specify how many patients were investigated outside of bout and for how long the patients had been attack-free [65]. All the patients included in the present study [51] were in active bout and we did not find a significant difference between prevalence of sleep apnea in patients compared with controls. However, patients did present more severe cases and a numerically higher apnea-hypopnea index (AHI), but it is entirely conceivable that this may have been caused by the massive over-representation of smokers in the patient group. Our patients were only investigated during the active part of the disorder and the possibility that hypothalamic dysregulation produces sleep apnea during the cluster period cannot be excluded. However, it seems unlikely that the apnea event itself is the trigger of nocturnal CH attacks, as no connection between the apnea events and nocturnal attacks were identified. Further anecdotal evidence provides no indication that treatment of the apnea alleviates the headaches [51, 65].

### Fewer arousals is a common finding in headache disorders

The role arousals play in healthy sleep is not completely clear but by definition they signify cortical activation. It has been suggested that they ensure the reversibility of the sleep stage and connect the sleeper with the (dangers of the) outside world [84]. They may represent a disrupting feature of sleep but may also be necessary for normal, healthy sleep and are associated with autonomic activation [84]. They are the result of changes in activating systems located in lower brain centers and the finding of reduced arousals – hypoarousal – in CH patients suggests reduced activity of ascending projections from the thalamus, hypothalamus and brainstem to the cortex. Hypoarousal has been identified before in other headache diagnoses [85–90] but the significance remains unknown. In the present study it is particularly surprising that the patients have fewer arousals, since they, in parallel with this finding, present with a higher AHI, traditionally associated with a tendency towards a higher number of (pathological) arousals. As with sleep in general, arousals may be affected by homeostatic pressure, and in this patient group it is unknown which role is played by underlying pathophysiological mechanisms and the direct influence of nocturnal CH attacks.

### Reduced hypocretin and trigeminal pain processing

Investigation into the physiological actions of HCRT is an area of active research, there are many unknowns and a discussion hereof remains somewhat hypothetical. It has, however, become clear that the phenotype exhibited in narcolepsy with cataplexy, brought about by a complete loss of hypocretinergic signaling, does not reveal all of HCRT’s functions. HCRT modulates the function of dopaminergic, histaminergic, noradrenergic and serotonergic neurons [91–93] in a complex manner and project widely in the CNS including to the LC, raphe nuclei, hypothalamic nuclei, spinally to the dorsal and ventral horns, to motor nuclei and limbic regions as well as the neocortex [94]. The dramatic effect of the complete loss of hypocretinergic signaling demonstrated in narcolepsy with cataplexy has given rise to a theory of a switching or gating function of HCRT in neuronal, including trigeminal, processing [93]. That HCRT plays a role in the modulation of trigeminal pain processing is supported by four lines of evidence:

1. Studies show an increased prevalence of migraine in narcolepsy patients [95, 96]. Further, the fact that dual HCRT antagonists frequently produce headache (although not migraine or CH-like pain) as a side effect [97, 98] have provided indirect evidence that hypofunction of HCRT signaling may destabilize trigeminal nociceptive processing resulting in headache.
2. Animal studies demonstrate that administered HCRT-1 produces anti-nociceptive results and HCRT-2 pro-nociceptive results [99]. Further, rats treated systemically with HCRT-1 have inhibited nociceptive responses of TNC neurons in response to electrical stimulation of the dura mater [100].
3. The HCRT-1 concentration has been shown to be increased in MOH and chronic migraine compared with healthy controls [101]. In CH, we found decreased levels of HCRT-1 (but within normal levels) which is in agreement with a prior small study which found numerically decreased levels in ECH but not CCH [102].
4. Genetic studies implicate the HCRT-system in CH; studies show that a particular polymorphism in the HCRT-2 receptor gene may increase the risk of CH [32, 34, 103], although not entirely consistently [35, 104].

Thus, clinical and animal studies suggest that HCRT may indeed play a fundamental role in the way the CNS processes pain, particularly cranial nociception. Whether the observed reduced levels of HCRT-1 levels in the CSF of CH patients truly reflects a hypofunction of hypothalamic descending antinociceptive signaling, or is simply an epiphenomenon, perhaps reflecting overall hypothalamic hypofunction, remains to be elucidated in future studies. HCRT-1 concentrations may be influenced by sleep macrostructure [105, 106] so mechanisms may be complex. However, as with testosterone, it is possible that this diminished release somehow alters pain-thresholds in the trigeminal complex. Further, hypocretinergic input to nuclei involved in autonomic control may be particularly interesting in this regard [107].

### Is autonomic dysregulation a purely local affair?

The cranial autonomic symptoms of CH are caused by an increase in parasympathetic outflow from the superior salivatory nucleus. This produces symptoms such as lacrimation and rhinorrhea. Dilatation of the internal carotid artery results in compression of the oculomotor nerve producing diminished sympathetic innervation resulting in ptosis and miosis. The different manifestations of these symptoms in CH patients most likely reflect a highly variant facial anatomy. While activation of the trigeminal autonomic reflex is undeniable, it is still unknown what triggers this activation. Additionally, involvement of systemic autonomic control in CH is the subject of debate. Based on previous observations, it is likely that central mechanisms involved in CH-pathology influence, or are influenced by, a dysfunction of autonomic control. One observational study showed that as many as 71 % of daytime attacks occur during physical relaxation [108] - a time of parasympathetic dominance. A few studies specifically investigating cardiovascular responses to various challenges in CH patients seem to indicate increased activity of the parasympathetic system [38, 48] although not entirely consistently [44, 45].

In the present results, significant changes in the HRV spectral and non-linear analysis of the response to tilt were found during HUT. The changes found would suggest a blunted sympathoexcitatory response to the change from supine to standing position. In the setting of (posterior) hypothalamic dysregulation, evidenced by radiological [23], endocrinological [27] and the present clinical findings of rhythmicity, dysregulation of the central hub of autonomic control located here (dorsomedial and paraventricular nucleus) [109], agrees with current theories of a central pathological mechanism. A previous study found an increased sympathoexcitatory drive during HUT in eight CCH patients after implantation of a DBS system [43]. Further, decreased levels of noradrenaline has been found in CH and could be related to clinical features [110].

Consequently, a pertinent question remains whether the observed blunted sympathoexcitatory response is an epiphenomenon of general hypofunction of hypothalamic nuclei, or if it reflects a fundamental pathological process. It has been theorized that diminished sympathetic drive may explain other findings in CH such as lower melatonin [73, 74, 111], lower testosterone [112–114], increased cortisol [115] (reviewed in [27]) and why manipulation of this axis with prednisone is effective in CH but not in other primary headache disorders [116]. Such a theory would need to address the unique features of CH in comparison to other primary headaches. However, findings of both sympathetic and parasympathetic hypo- and hyperfunction in migraine may be contradictory [117–120]. No consensus exists in migraine and different methods and inherent differences between migraineurs and CH patients (age, sex, smoking habits) make the comparison complex.

Interestingly, patients suffering from narcolepsy with cataplexy, who are completely HCRT-deficient, do not respond to tilt with an increase in LFnu, a decrease in HFnu, and increased LF/HF as do normal controls [121]. This suggests that HCRT may be necessary for the increase in sympathetic tone necessary for a transition to sympathodominant balance of autonomic tone after tilt, and may suggest a connection between the present findings of a weakened sympathodominant response to HUTT and reduced HCRT-1 levels. Speculatively, in CH-predisposed individuals, physiological changes in autonomic tone may contribute to disinhibition of nociceptive processing in the TNC in turn resulting in unrestrained activation of the trigeminal autonomic reflex.

### Methodological considerations

The present studies present data from the largest, best-characterized population of CH patients with specialist-verified diagnoses. The data is of a high quality and proven and validated methods were applied. By contacting the patients personally a high degree of precision could be obtained and ambiguities resolved. It is further strengthened by the fact that several investigations were carried out in the same population. We used a novel way of characterizing the total headache load by means of an index, as the current diagnostic criteria have some shortcomings. The clinical investigations were conducted in-hospital which provided ideal conditions for observation.

CH presents some unique challenges when it comes to studying the attacks. Because of the extreme pain and ethical considerations both acute and preventive medications were allowed during the investigations. In the three clinical studies analyses were conducted to identify a possible influence of this on results, but none was found. Nevertheless, it cannot be ruled out that it may have affected results. Further, it was noticed that the patients suffered noticeably fewer attacks while admitted. This is an interesting finding, as it implies that the increased sympathetic tone associated with a stay in new surroundings may affect the generation of cluster attacks. The investigators had expected this to a certain degree and allowed for ample time for the patient to familiarize him- or herself with the department. Further, the patient stayed in a quiet, single room with television and internet access and PSG was conducted on two nights to allow the patient to become accustomed. For more detailed methodological considerations please see [28, 51, 52, 63].

## Conclusions

Overall the present results support a causative role or very intricate involvement of central mechanisms in the pathogenesis of CH. However, due to the complex interactions and overlap of anatomy and physiology, there is considerable difficulty associated with determining the contributions of structures involved. It may be possible that some of the observed changes are epiphenomena reflecting other central processes of brainstem and hypothalamic circuits.

It is tantalizing to theorize that the changes in the autonomic system and hypocretinergic signaling contribute to the stereotypical presentation of the CH patient. At the root of these mechanisms lie changes in hypothalamic and brainstem nuclei which are detrimental to the normal regulation of sleep, headache or no headache. In the setting of a weakened entrainment of the biological clock, and dysregulation of the posterior hypothalamus, the balance of nociceptive and antinociceptive input to the trigeminal nucleus is dysregulated, resulting in disinhibition of the trigeminal autonomic reflex: A downward, self-perpetuating spiral of pain and activation of the autonomic reflex. As the reflex runs its course, and is eventually exhausted, the patient is left for want of pain relief, living in constant fear of when the next attack will arise. As the illustrious Nicolaes Tulp noticed several hundred years ago: The unfathomable pain and predictability of the CH attacks become an inseparable part of the patients’ lives.

## Future perspectives

Future endeavors in CH research should focus on the following aspects of the disorder:

1. Prospective investigations of sleep and chronobiology in CH patients in the time leading up to a cluster may provide valuable insight into the changes taking place. It is possible that changes in hypothalamic nuclei manifest themselves before the breakthrough of cluster attacks, possibly as changes in sleep regulation.
2. Further investigation of specific sleep-related phenomena may provide insight into the mechanisms affected in CH, especially the investigation of sleep outside of the cluster. Careful attention should be paid to how much time has passed since the last attack, i.e. how far outside of the cluster the patient actually is.
3. Manipulation of the trigeminal autonomic reflex is a promising therapeutic target. It is possible that feedback mechanisms may influence the function of brainstem nuclei and this interaction should be investigated.

## Abbreviations

BMI: Body-mass index (BMI = kg/m^2^)
BP: Blood pressure
CH: Cluster headache
CCH: Chronic cluster headache
CNS: Central nervous system
CSF: Cerebrospinal fluid
DBP: Diastolic blood pressure
DBS: Deep brain stimulation
ECG: Electrocardiogram
ECH: Episodic cluster headache
HCRT: Hypocretin (orexin)
HF: High frequency (n.u.: normalized units)
HR: Heart rate
HRV: Heart rate variability
HUT: Head-up position
HUTT: Head-up tilt table test
LC: Locus coeruleus
LF: Low frequency (n.u.: normalized units)
LM: Limb movements
MEQ: Morningness-Eveningness Questionnaire
MSLT: Multi-sleep latency test
PLM: Periodic limb movements
PAG: Periaquaductal grey
PG: Pterygopalatine (sphenopalatine) ganglion
PSG: Polysomnography
PSQI: Pittsburgh Sleep Quality Index
SBP: Systolic blood pressure
SCN: Suprachiasmatic nucleus
SDNN: Successive normal beats
SSN: Superior salivatory nucleus
REM: Rapid eye movement
TAC: Trigeminal autonomic cephalalgia
TG: Trigeminal ganglion
TNC: Trigeminal nucleus caudatus
